# Impact of incidental synucleinopathy in mild cognitive impairment due to Alzheimer disease

**DOI:** 10.1093/jnen/nlae009

**Published:** 2024-02-12

**Authors:** Jahnavi Shriram, Michael Malek-Ahmadi, Chase Irwin, Marwan Sabbagh

**Affiliations:** University of Arizona College of Medicine, Phoenix, Arizona, USA; Department of Biomedical Informatics, University of Arizona College of Medicine, Phoenix, Arizona, USA; Department of Biostatistics, University of Arizona College of Medicine, Phoenix, Arizona, USA; Department of Neurology, Barrow Neurological Institute, Phoenix, Arizona, USA

**Keywords:** Alzheimer disease, Mild cognitive impairment, Mixed synucleinopathy

## Abstract

Recent evidence suggests that the presence of α-synuclein Lewy bodies (LBs) correlates with accelerated disease progression in patients with Alzheimer disease (AD) but it is unclear whether this effect is also exerted in the mild cognitive impairment (MCI) phase of AD. We sought to determine whether incidental LB pathology in patients with MCI due to AD is associated with a faster rate of cognitive decline compared to MCI controls without LB pathology. We identified patients within the National Alzheimer’s Coordinating Center (NACC) database with MCI due to AD and stratified the cohort by the presence or absence of synucleinopathy. We utilized a repeated measures longitudinal analysis of Mini-Mental State Examination (MMSE) scores to determine whether the decline in performance occurred at a greater rate in the synucleinopathy patients. A total of 206 participants were studied; 80 had coincident synucleinopathy. The rate of decline in MMSE scores between the groups did not differ. This may suggest that a synergistic effect of LB and AD neuropathology is only appreciable in the later stages of disease progression. Further investigation into the effect of mixed LB and AD pathology in the early stages of cognitive impairment is warranted to highlight opportunities for targeted early intervention in patients.

## INTRODUCTION

Dementia due to Alzheimer disease (AD) is the leading cause of dementia worldwide. In the United States alone, over 6.5 million older adults have been diagnosed with AD as of 2021, a number expected to triple by 2060 (1). AD is characterized by progressive impairments in memory, judgment, and reasoning.

The hallmark neuropathologic features of AD include amyloid plaques and tau neurofibrillary tangles. The density of these features can be quantified using neuroimaging and these measures have been shown to correlate with the severity of symptomatic burden in patients with AD (2).

Recent evidence suggests that the co-occurrence of neuropathologies associated with other forms of dementia also correlates with disease severity in individuals with AD (3–8). Many older adults harbor “mixed pathologies,” wherein multiple neurodegenerative and cerebrovascular pathologic markers are present. This is commonly observed as amyloid plaques and tau tangles indicative of AD co-occurring with microinfarcts and white matter changes of cerebrovascular disease. Mixed pathologies exert independent but combined effects to lower the threshold for developing cognitive decline and clinically overt dementia (9).

Lewy bodies (LBs) are pathologic variants of the α-synuclein protein associated with both Parkinson disease and Lewy body dementia (LBD). It has been well documented in the literature that patients with dementia due to AD and co-occurring LB pathology showed faster rates of cognitive decline than those without synucleinopathy. As far back as 1998, a Consortium to Establish a Registry of Alzheimer’s Disease (CERAD) study of autopsy-confirmed patients with AD showed that those with at least one or more LB identified on neuropathologic examination had a higher mean rate of decline in Mini-Mental State Examination (MMSE) score than those with no synucleinopathy (6). A study of the University of Washington’s AD patient registry similarly showed that the rate of MMSE score decline was greater in patients with AD and LB inclusions (4). In addition to MMSE score decline, patients with AD and LBs have demonstrated a shorter time to development of symptoms such as diurnal hypersomnia, depression, and institutionalization (5).

More recently, a 2017 National Alzheimer’s Coordinating Center (NACC) study compared rates of decline within 4 cognitive domains and found that the increased rate of decline seen in AD + LB patients was pronounced in the domains of attention/working memory and executive function (3). A 2019 study further delineated coincident synucleinopathy as meeting density thresholds for concurrent diagnosis of LBD and those with subdiagnostic thresholds that were classified as unspecified diffuse LB disease (7). Both AD + LBD, as well as AD + LB disease cohorts, showed significantly greater MMSE decline slopes compared to the AD-only cohort (7). Finally, a 2023 study showed a faster rate of global cognitive decline in biomarker-identified AD + LB groups compared to pure AD, pure LB, and AD−/LB− groups including participants in both mild cognitive impairment (MCI) and overt dementia disease stages (8).

While the impact of synucleinopathy on AD progression has thus been described, recent evidence indicates that curtailing the disease burden of AD is likely more effective prior to the onset of overt dementia, when cognitive changes impair or prevent patients’ daily independent functioning. The preclinical stages of AD precede the onset of symptoms by decades. Cognitively unimpaired patients may have early pathologic signs of neuroimaging in the absence of measurable cognitive impairment. Cognitively unimpaired patients may progress to reporting subjective memory concerns, despite continuing to show normal cognitive functioning in assessments such as the MMSE. The final, prodromal stage is MCI, in which individuals display measurable deficits of cognitive function that yet do not meet the criteria for a diagnosis of AD. Both subjective memory concern and MCI status confer a higher risk of developing dementia due to AD (10–12).

As a result, there has been a recent effort to investigate molecular processes that contribute to the preclinical and prodromal stages of AD progression to identify potential early diagnostic and therapeutic targets. With the mounting evidence that co-occurring LB accelerates the clinical deterioration of AD, it is thus warranted to investigate whether incidental synucleinopathy also hastens disease progression in prodromal AD. In a prior comparison of cognitive domain score decline in patients without clinical dementia at baseline, the discrepancy between participants thought to have MCI due to AD with LB pathology compared to MCI due to AD alone was comparable to that of the analogous cohorts with diagnosed dementia (3). Beyond this finding, the prodromal impact of co-occurrent LB has not been extensively studied.

We sought to determine whether the presence of incidental synucleinopathy is associated with a higher rate of decline in cognitive performance among patients in the MCI stage of AD. We hypothesize that people with MCI due to AD who have incidental synucleinopathy have a more rapid rate of cognitive decline than those with MCI due to AD that do not show synucleinopathy.

## MATERIALS AND METHODS

### Data source

Data for this investigation were extracted from the Unified Data Set (UDS), Neuropathology dataset (NP), and Genetic data (Gen) of the NACC database. This database was established by the National Institute on Aging (www.nia.nih.gov). Since 1984, the NACC has received longitudinal data from all Alzheimer’s Disease Research Centers (ADRCs) across the United States. These data include diagnostic, demographic, and clinical evaluations of patients with dementia from multiple visits that can be used to trace cognitive decline. The NACC’s UDS was established in 2005 and prospective data with clinical evaluations of individuals are now collected annually. The data from each individual are obtained from trained clinicians or personnel in the form of an office meeting, home visit, or telehealth visit which take place on an annual basis. Our analysis used data from UDS visits conducted between September 2005 and February 2022, taking place at 46 ADRCs.

### Standard protocol approvals, registrations, and patient consents

All participants who have data in the NACC database were enrolled at ADRCs nationwide. All participants provided informed consents at the time of enrollment that were approved by their respective institutional review boards. The informed consent included permission for deidentified data to be uploaded to and included in the NACC database for use in research by qualified investigators. No additional patient informed consent was required for this individual retrospective observational case-control study.

### Selection and description of participants

Our participants were selected on the basis of having a primary etiologic diagnosis of AD and a clinical diagnosis of MCI for at least 2 subsequent annual NACC visits. In v.3 of the UDS, a clinician determines that the patient does not have dementia based on the McKhann et al criteria (13), and confers a diagnosis of MCI based on the Albert et al criteria (14). MMSE scores are not included in the Albert definition of MCI (14). Primary etiologic attribution of MCI to AD was determined using a standardized probabilistic framework.

We stratified the participants into cases with incidental LBs (MCI/AD LB+) and controls who had no synucleinopathy (MCI/AD LB−). In the NP dataset, patients positive for synucleinopathy are identified on brain autopsy by the presence of LBs in one or more brain regions including the brainstem, limbic system/amygdala, neocortical, or olfactory bulb/unspecified. While the NP subcategorizes LB pathology based on predominant brain region, we grouped all subcategories into a single categorical variable of positive (LB+) or negative (LB−) synucleinopathy due to the impractically of small sample sizes of subdivided LB groups. We defined incidental synucleinopathy as LB+ participants who did not carry a concurrent etiologic diagnosis of MCI due to LB.

Participants were excluded if they were found to carry non-AD or LB comorbid neuropathologies on brain autopsy, including frontotemporal lobar degeneration subtypes, vascular dementia using the Román definition (15), traumatic brain injury, and others. A comprehensive list of excluded comorbidities can be found in Supplementary Data Table S1. Participants were grouped into cases with positive synucleinopathy (MCI/AD LB+) and pure AD controls (MCI/AD LB−).

### Statistical analysis

Descriptive statistics were stratified by LB status and reported as frequencies and proportions or means and standard deviations. Chi-square tests or Fisher exact tests and 2 sample t-tests were used to evaluate differences in strata across descriptive variables. Comparisons included age at baseline, years of education, race, sex, Braak stage of AD pathologic severity assessed on autopsy, and proportion of *APOE* ε4 allele carriers. Patients are considered ε4 allele carriers if their genotype includes at least 1 copy of the ε4 allele.

For our primary outcome, global MMSE scores were used to assess the rate of cognitive decline for LB+ and LB− patients from a starting point of their baseline visit, defined as their first NACC encounter with a clinical diagnosis of MCI, for up to 5 annual follow-ups (6 total visits). We employed an adjusted, linear mixed-effect model and performed between-group comparisons by fitting the interaction between the comparison group (LB+ or LB−) and time point (baseline or follow-up visit) as fixed effects and patient identity as the random effect. We also reported the estimated decline within-group for up to 5 follow-up visits. For each given follow-up period, we performed a complete case analysis and reported the available sample size not lost to follow-up. Our model controlled for patient demographics including age at baseline, years of education, race, sex, and *APOE* genotype. For our secondary outcomes, we assessed the time to dementia and time to death for our cohort by LB status. For each outcome, we plotted the Kaplan-Meier curve and reported the median survival time and its corresponding 95% CI. We compared survival curves across comparison groups using the log-rank test and reported the p-value. For all analyses, an alpha level of 0.05 was used to determine statistical significance.

The data analysis for this article was generated using SAS software, Version 9.4 of the SAS System for Windows (SAS Institute Inc., Cary, North Carolina). All plots were created using R statistical software v4.3.1.

### Data availability

Data used for this study are made publicly available through the NACC.

## RESULTS

### Descriptive analysis

A total of 126 MCI/AD LB− controls and 80 MCI/AD LB+ cases met inclusion criteria and were included in the analysis (Supplementary Data Fig. S1). LB+ cases were younger at baseline (p = 0.002) and had a higher proportion of ε4 carriers (p = 0.001) than LB− controls (Table 1). Otherwise, LB+ cases and LB− controls were similar in sex (p = 0.46), race (p = 0.32), and years of education (p = 0.54) (Table 1).

**Table 1.** Characteristics of MCI/AD cases with and without Lewy body pathology

	Controls (MCI/AD LB−) n = 126	Cases (MCI/AD LB+) n = 80	**p value** *
Baseline age, mean (SD)	80.43 (8.83)	76.05 (10.59)	0.002^†^
Sex, n (%)			0.46
Male	77 (61.11)	53 (66.25)	
Female	49 (38.89)	27 (33.75)	
Race, n (%)			0.32
Caucasian	118 (93.65)	78 (97.50)	
African American	7 (5.56)	1 (1.25)	
Asian	1 (0.79)	1 (1.25)	
Years of education, mean (SD)	15.40 (3.16)	15.68 (3.25)	0.54
APOE ε4 carrier, n (%)	47 (37.30)	48 (60.00)	0.001^†^
Alzheimer’s Braak stage, n (%)			0.12
0—Stage 0	1 (0.80)	0 (0.00)	
1—Stage I (B1)	5 (4.00)	2 (2.50)	
2—Stage II (B1)	20 (16.00)	4 (5.00)	
3—Stage III (B2)	12 (9.60)	5 (6.25)	
4—Stage IV (B2)	24 (19.20)	15 (18.75)	
5—Stage V (B3)	26 (20.80)	17 (21.25)	
6—Stage VI (B3)	37 (29.60)	37 (46.25)	
Unknown	1 (0.80)	0 (0.00)	
Lewy body distribution			—
Brainstem	—	4 (5.00)	
Limbic or amygdala	—	47 (58.75)	
Neocortical	—	22 (27.50)	
Unspecified	—	7 (8.75)	

* Two-sample t-test for continuous variables; Chi-square or Fisher exact test for categorical variables.

† Indicates statistically significant difference for alpha level = 0.05.

To compare the severity of AD pathology between the 2 groups, we reported on the distribution of Braak scores for >99% (n = 205) of our cohort. The majority of participants in both groups were found to be in Braak stages V and VI, in which neurofibrillary degeneration involves the neocortex (16). The proportion of participants in each Braak stage was similar between LB+ cases and LB− controls. In LB+ cases, the majority had a limbic or amygdala-predominant distribution of LBs.

### Mixed-model longitudinal analysis

**Figure 1.**
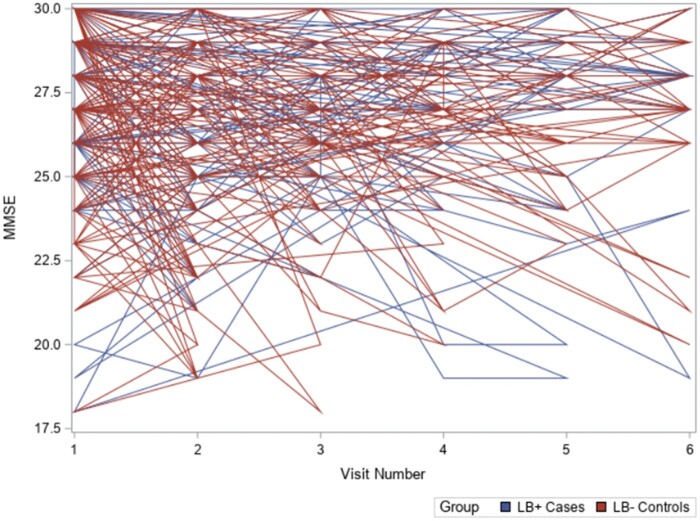
Whole-cohort MMSE score trends across Visits 1–6.

Collective MMSE score trends across the whole cohort are shown in Figure 1. Table 2 summarizes the results from our longitudinal analysis. Both cohorts experienced substantial loss to follow-up by the second post-baseline visit (Table 2). At baseline, LB+ cases mean MMSE scores (26.46, 95% CI: 25.31–27.62) were comparable to LB− controls (26.58, 95% CI: 25.49–27.66). Importantly, MMSE scores are not included in the Albert 2011 criteria (14) used to diagnose MCI, which accounts for the high baseline MMSE scores of our cohort. When evaluating within-group changes from baseline, LB+ cases only saw a significant decrease when comparing baseline to their first follow-up visit (−0.68, 95% CI: −1.16 to [−0.20]). LB− controls saw a within-group significant decrease in mean MMSE scores compared to baseline on all 5 post-baseline visits. No significant differences were detected when evaluating the change in mean MMSE scores between LB+ cases and LB− controls for all 5 post-baseline visits (Table 2).

**Table 2.** Absolute change in MMSE scores from baseline*

	Sample size at visit		Mean MMSE (95% CI)		Mean change from baseline to visit (95% CI)			
	LB−	LB+	LB−	LB+	LB−	LB+	Difference in change^†^	p value^‡^
Baseline	n = 126	n = 80	26.58 (25.49–27.66)	26.46 (25.31–27.62)	—	—	—	—
Visit 2	n = 125	n = 76	26.20 (25.11–27.28)	25.79 (24.63–26.94)	−0.38 (−0.76–0.00)	−0.68 (−1.16–[−0.20])	−0.30 (−0.91–0.31)	0.34
Visit 3	n = 64	n = 45	25.58 (24.44–26.72)	26.29 (25.09–27.50)	−1.00 (−1.58–[−0.41])	−0.17 (−0.88–0.54)	0.82 (−0.09–1.75)	0.08
Visit 4	n = 34	n = 28	25.41 (24.17–26.65)	26.22 (24.94–27.50)	−1.17 (−1.96–[−0.37])	−0.25 (−1.16–0.67)	0.92 (−0.29–2.13)	0.14
Visit 5	n = 26	n = 20	25.43 (24.11–26.76)	25.80 (24.39–27.20)	−1.14 (−2.08–[−0.21])	−0.67 (−1.76–0.42)	0.47 (−0.96–1.91)	0.52
Visit 6	n = 19	n = 11	25.24 (23.81–26.67)	25.56 (23.93–27.20)	−1.34 (−2.43–[−0.25])	−0.90 (−2.29–0.49)	0.44 (−1.33–2.20)	0.62

* Complete-case analysis was performed for each given follow-up period. Difference in change between groups are marginal differences (95% CI) estimated from mixed-effects models.

† Calculated as (ΔLBD[+] – ΔLBD[−]).

‡ p-Value for between-group comparisons.

### Time-to-event analyses

**Figure 2.**
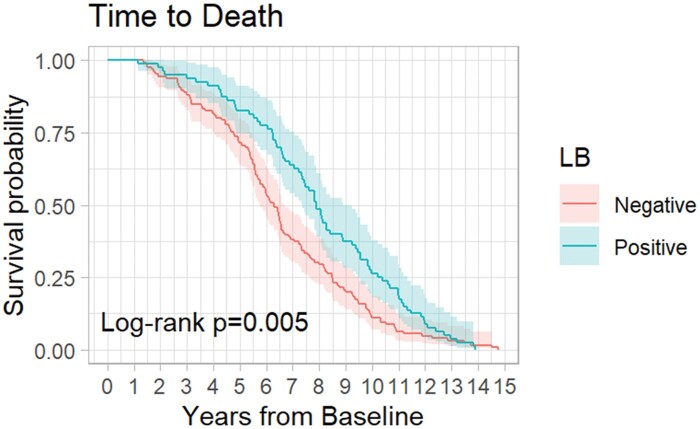
Time-to-death Kaplan-Meier curve.

The Kaplan-Meier curves for LB+ cases and LB− controls time until death are presented in Figure 2. We obtained data on all patients’ time of death; therefore, none were lost to follow-up. LB+ cases had a longer median survival time (7.88, 95% CI: 7.36–8.89) than LB− controls (6.29 years, 95% CI: 5.77–6.72). The log-rank test indicated that the survival curves were different between groups (p = 0.005).

**Figure 3.**
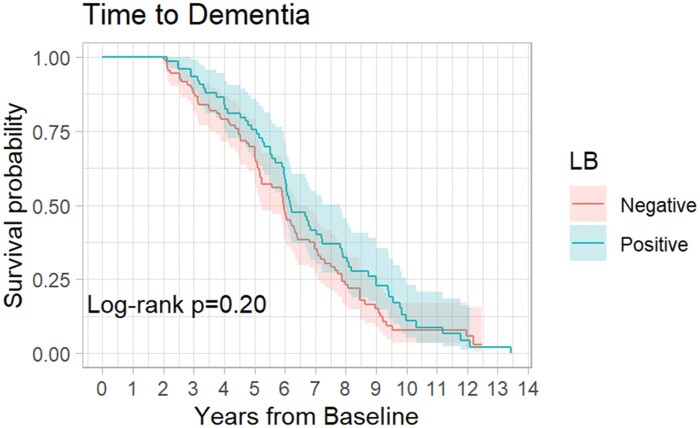
Time-to-dementia Kaplan-Meier curve.

The Kaplan-Meier curves for LB+ cases and LB− controls’ time until dementia are presented in Figure 3. About 65 LB+ cases and 86 LB− controls received a diagnosis for dementia before being lost to follow-up. LB+ cases had a median time to dementia of 6.18 years (95% CI: 5.98–7.81) while LB− controls had a median time to dementia of 5.95 (95% CI: 5.25–6.42). The log-rank test indicated that the survival curves were not different between groups (p = 0.20).

## DISCUSSION

Currently, AD research emphasizes investigation into early pathological and subtle cognitive performance changes occurring up to decades prior to overt symptomatology (10–12). This is due to discoveries highlighting that the most potent window for meaningful intervention into AD progression is in the MCI phase prior to patients meeting diagnostic criteria for clinical dementia (17). Additionally, recent studies have highlighted the influence of mixed neuropathologies related to other dementia etiologies influencing disease course in patients with dementia due to AD (3–8). In particular, the presence of synucleinopathy, typically characteristic of dementia due to LB, has been shown to accelerate cognitive decline in patients with a primary diagnosis of AD. This effect has been demonstrated even in AD patients with incidental LBs at a density below the threshold for diagnosis of concurrent LBD (7).

We sought to investigate whether AD patients who ultimately developed synucleinopathy detectable on autopsy showed similarly accelerated disease progression in the MCI phase of the disease course, prior to meeting the criteria for dementia. Finding a significant effect of mixed pathology in prodromal AD would suggest that investigation into early intervention therapeutics should take subthreshold mixed pathologies into account. We hypothesized that patients having MCI due to AD with incidental synucleinopathy would have a faster rate of decline in cognitive performance over longitudinal clinical encounters compared to patients with pure MCI due to AD. To compare the rate of cognitive decline in LB+ cases compared to LB− controls, we performed a longitudinal mixed-methods analysis of MMSE scores over consecutive annual clinical encounters captured in the NACC database.

Our cohort consisted of 126 LB− controls and 80 LB+ cases that had complete demographic, neuropathologic, genetic, and MMSE data for at least 2 consecutive annual clinical encounters while in the MCI phase of AD. Our mixed-model regression comparing the rate of change in MMSE scores among LB− controls and LB+ cases over consecutive NACC visits included baseline age, years of education, race, sex, and *APOE* genotype as covariates. Overall, we found that there was no difference in the rate of MMSE score change between controls and cases. Between Visits 1 and 2, MMSE scores decreased by 0.30 points more in the LB+ group compared to controls, but this result was not significant. In addition, MMSE scores of LB+ cases decreased by 0.48 points less than controls between Visits 1 and 6. This difference in rate was also not significant, though this comparison was limited by the dwindling sample size of participants with complete MMSE score data in both cases and controls in later Visits.

To gain a better understanding of how decline in MMSE scores varied based on LB status, we estimated differences in MMSE scores over time independently in cases and controls. We found that both groups saw a significant decrease in MMSE scores at their second visit compared to their first visit. Furthermore, our control group continued to see a significant cumulative decrease in MMSE scores compared to baseline at Visits 3, 4, 5, and 6. This was not seen in the LB+ cases, likely due to the diminishing sample size beyond Visit 2.

We discovered baseline differences in the characteristics of our comparison groups. First, our control group was older at their first NACC visit compared to LB+ cases. Since older age strongly correlates with the severity of AD, these results suggest that at baseline, controls may have been farther along in the disease course compared to cases. However, the proportion of participants in each Braak stage assessed on autopsy did not differ between the 2 groups. Furthermore, it is unknown whether the rate of decline in cognitive performance accelerates with AD progression or its known correlates; i.e. whether the slope of cognitive decline in AD is linear or nonlinear over the disease course. Thus, a significant difference in baseline age likely did not influence our comparison of the rate of cognitive decline between experimental groups.

There was a significantly higher proportion of *APOE* ε4 allele carriers in our LB+ group compared to controls. Since the ε4 allele is known to accelerate AD progression, this difference suggests that the LB+ group was predisposed to a faster rate of cognitive decline compared to controls through a mechanism independent of mixed neuropathology. Our mixed-model analysis controlled for ε4 allele carrier frequency in order to mitigate this potential bias.

In addition to MMSE score change, we investigated whether secondary outcomes including time to development of dementia and time to death differed between controls and LB+ cases using Kaplan-Meier log rank curves. LB− controls had a shorter median survival time compared to LB+ cases. This may be attributable to lead time bias, as the control group was older at baseline compared to cases. We found no difference in time before meeting the criteria for dementia across both groups.

Our finding that the rate of decline in MMSE scores did not differ between MCI/AD patients with and without incidental synucleinopathy is consistent with the principle that neuropathologic changes precede clinical cognitive performance impairment. However, these findings deviate from prior studies showing that the presence of incidental LBs is correlated with accelerated cognitive decline in patients with AD (3–4, 6–8). Importantly, prior studies have included patients meeting the criteria for dementia, rather than restricting comparisons to the MCI phase. This may account for the deviation of our results, implying that there is a threshold after which mixed pathologies exert a synergistic effect accelerating disease progression.

Disparate spatial distribution of neuropathology is one possible explanation of why early mixed pathologic changes may not exert an interactive effect. A previous comparison of patterns of cortical thinning in patients with pure AD, pure LB spectrum disease, and mixed AD/LB disease found no significant interactive effect of concomitant AD and LB pathology in any brain region (18). However, the closest overlap in regions that were independently affected by each disease occurred in the parietal cortex. Furthermore, AD/LB mixed pathology exerted a significant interactive effect on visuospatial cognitive dysfunction as captured by the Rey-Osterrieth Complex Figure Test, which may correspond to overlapping effects of AD and LB pathology in the parietal visual association cortices. These results may also imply that as mixed disease progresses, neuropathologic distribution becomes more widespread, such that regions of overlap between neuropathologies also increase. Thus, increasing spatial overlap between simultaneous neuropathologies may account for greater synergy in effecting cortical damage only after considerable disease progression, i.e. when clinical dementia becomes overt.

Our study is limited by small sample size, particularly in the density of participants with complete clinical data later than 2 years after the initial presentation. Since the preclinical course of AD is known to occur over decades, more meaningful differences in rate of change between LB− controls and LB+ cases may be seen with a longer time scale of data. In addition, previous literature suggests that the Clinical Dementia Rating scale (CDR) Dementia Staging Instrument may be a more sensitive metric of cognitive performance in mild stages of dementia (19). Further investigation of the influence of mixed pathology in MCI should utilize this and other cognitive performance metrics to gain a more holistic picture of subtle differences in cognitive decline. This is especially warranted given previous findings that incidental synucleinopathy is correlated with accelerated decline in the specific cognitive domains of attention/working memory and executive function (3, 18). Similarly, in light of evidence that spatial distribution of neuropathologies may influence their interactive effect on dementia-related brain morphological changes (18), further investigation should include regionality of synucleinopathy and amyloid/tauopathy as a covariate.

In addition, CSF seed amplification has recently become available as a biomarker of synucleinopathy. Together with new biomarkers of amyloid and tau that can be used to support the diagnosis of AD, this presents an opportunity to prospectively study differential rates of clinical deterioration from the point of detection of synucleinopathy. This approach has been validated in a study demonstrating that patients with positive CSF biomarkers of pathologic synuclein, amyloid, and tau showed a more rapid global cognitive decline compared to participants with solely LB or AD-specific biomarkers, in a cohort consisting of both MCI and dementia patients (8). Utilizing this approach in a cohort restricted to patients in the MCI stage can further contextualize our findings to shed light on the early-stage effects of mixed pathology.

In conclusion, we sought to reconcile two emerging paradigms in AD: that of the critical opportunity for intervention in the MCI phase and the influence of mixed neuropathologies in cognitive decline. We did not find that patients with mixed neuropathologies showed a higher rate of cognitive decline in the MCI phase compared to patients with only AD pathology. However, further comparison of decline in specific cognitive domains and discrete brain regions may be warranted to characterize the early effects of mixed pathology. This may highlight opportunities for targeted early intervention in patients with AD-related cognitive impairment.

## Supplementary Material

nlae009_Supplementary_Data
