# Supplementary material for: Impact of incidental synucleinopathy in mild cognitive impairment due to Alzheimer disease
Source: J Neuropathol Exp Neurol. 2024 Feb 12;83(4):230–7. doi: 10.1093/jnen/nlae009 (PMC10951969; doi:10.1093/jnen/nlae009)
Supplement: nlae009_Supplementary_Data [file nlae009_supplementary_data.zip › nlae009_Supplementary_Data/Supplemental Table 1.docx]

**Supplemental Table 1.** Excluded neuropathologic comorbidities

| **Excluded Neuropathologic Comorbidities** |  |
| --- | --- |
| Hippocampal sclerosis (CA1 and/or subiculum) |  |
| Medial temporal lobe sclerosis present (including hippocampal sclerosis) |  |
| FTLD with tau pathology (FTLD-tau) or other  tauopathy |  |
| ARTAG pathology |  |
| Pigment-spheroid degeneration/NBIA |  |
| Multiple system atrophy |  |
| Prion disease |  |
| Trinucleotide disease (Huntington disease,  SCA, other) |  |
| Malformation of cortical development |  |
| Metabolic/storage disorder of any type |  |
| WM disease, leukodystrophy |  |
| WM disease, multiple sclerosis or other  demyelinating disease |  |
| Contusion/traumatic brain injury of any type, acute |  |
| Contusion/traumatic brain injury of any type, chronic |  |
| Neoplasm, primary |  |
| Neoplasm, metastatic |  |
| Infectious process of any type (encephalitis,  abscess, etc.) |  |
| Herniation, any site |  |
| Down syndrome |  |
| Other pathologic diagnosis |  |
