# Supplementary figures and images for: Impact of incidental synucleinopathy in mild cognitive impairment due to Alzheimer disease
Source: J Neuropathol Exp Neurol. 2024 Feb 12;83(4):230–7. doi: 10.1093/jnen/nlae009 (PMC10951969; doi:10.1093/jnen/nlae009)

Supplemental Figure 1. Selection of participants.

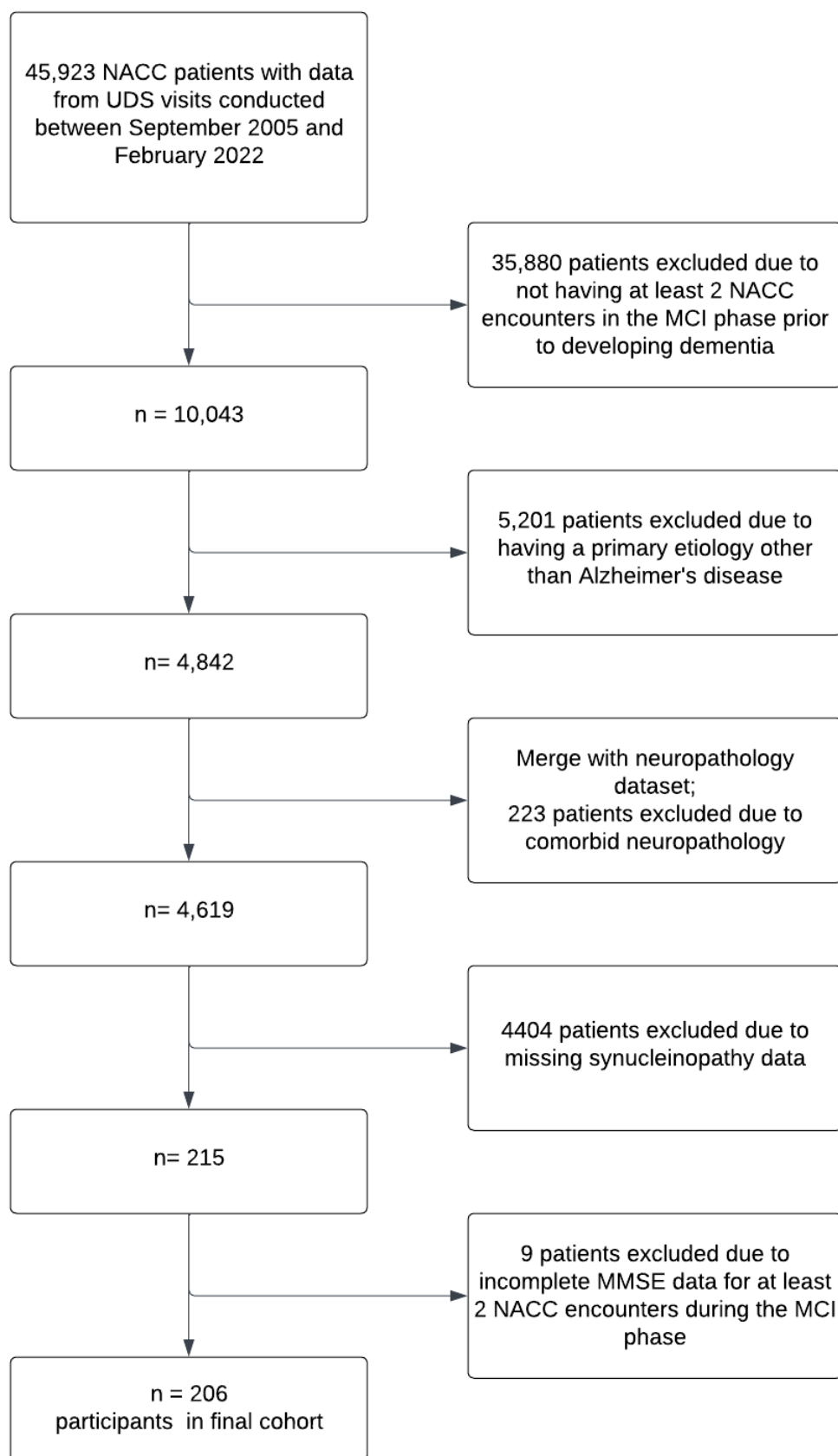

Supplement: nlae009_Supplementary_Data [file nlae009_supplementary_data.zip › nlae009_Supplementary_Data/Supplemental Figure 1.pdf]
